# CSF Biomarkers Profile in CADASIL—A Model of Pure Vascular Dementia: Usefulness in Differential Diagnosis in the Dementia Disorder

**DOI:** 10.4061/2010/959257

**Published:** 2010-08-18

**Authors:** Patrizia Formichi, Lucilla Parnetti, Elena Radi, Gabriele Cevenini, Maria Teresa Dotti, Antonio Federico

**Affiliations:** ^1^Department of Neurological, Neurosurgical and Behavioural Sciences, University of Siena, 53100 Siena, Italy; ^2^Department of Neuroscience, Memory Clinic Alzheimer Centre, University of Perugia, 06123 Perugia, Italy; ^3^Department of Surgery and Bioengineering, University of Siena, 53100 Siena, Italy

## Abstract

Cerebral Autosomal Dominant Arteriopathy with Subcortical Infarcts and Leukoencephalopathy (CADASIL) is considered a model of pure vascular dementia (VD) because it occurs in young adults unlikely to have concomitant age and Alzheimer's Disease-(AD-) related pathology. CSF levels of *β*-amyloid 1-42 (A*β*42), total tau protein (t-tau), and phosphorylated tau-protein (p-tau), well accepted biomarkers of AD, were evaluated in 10 CADASIL patients, 22 AD patients, and 17 healthy age-matched subjects. Innotest *β*-amyloid 1-42, Innotest hTAU-Ag, and Innotest Phospho-tau 181p sandwich enzyme-linked immunoassay were used to determine CSF biomarkers levels. A case-control statistical analysis was carried out. CSF A*β*42 levels were significantly lower in CADASIL patients and considerable overlap with AD whereas t-tau and p-tau levels were normal and significantly different with respect to AD. A significant altered CSF biomarkers profile in a pure VD supports the use of CSF A*β*42, t-tau, and p-tau levels in the differential diagnosis of VD and AD.

## 1. Introduction

Despite the increased use of widely accepted diagnostic criteria over the last 1-2 decades, the differential diagnosis between Alzheimer disease (AD) and vascular dementia (VD), the most common causes of dementia in the elderly, is not always easy in the clinical practice. Many cognitively impaired patients, with a progressive history of classical cortical dementia, show different degrees of concomitant vascular lesions [1]. Vascular comorbidity may be present in 30%–60% of AD patients [2], and, conversely, AD pathology may be present in 40%–80% of VD patients [3], thus hindering diagnosis accuracy. Further, because the prevalence of both AD and VD increases with age, the coexistence of AD and VD in the elderly patients would also be expected to occur. 

Cerebral Autosomal Dominant Arteriopathy with Subcortical Infarcts and Leukoencephalopathy (CADASIL) is an inherited microvascular disease associated with a wide range of symptoms including migraine, mood disorders, and recurrent subcortical TIA/strokes [4, 5]. Two thirds of CADASIL patients early develop subcortical dementia, usually in the fourth-fifth decades of life [6]. Due to its monogenic nature and occurrence in young adults, CADASIL is considered a useful model of VD, in whom age- and AD-related pathology unlikely to coexist [7]. 

Beta-amyloid_1-42_ (A*β*42), total tau (t-tau), and phospho-tau (p-tau) proteins, indices of amyloid deposition, axonal damage, or tangle formation respectively, have been suggested as biomarkers for the diagnosis in dementia disorders [8]. The CSF profile of these proteins in patients with AD is characterized by decreased A*β*42 and increased t-tau and p-tau levels [9, 10]. On the contrary in VD, studies on these CSF biomarkers showed conflicting results: t-tau levels have been reported to be increased [11–13], normal [14, 15] or intermediate [16, 17], but in any case much lower than in AD; A*β*42 CSF levels in VD have been reported to be moderately decreased [18] or significantly overlapping with AD [19]. Few works deal with p-tau reporting either normal [20] or increased CSF levels [21] in VD. 

Since previous studies showed contradictory results in VD, we evaluated CSF biomarkers in CADASIL, a young-onset monogenic disease which offers a unique opportunity to define CSF biomarkers profile in a pure VD. For this purpose we assessed CSF A*β*42, t-tau, and p-tau levels in ten CADASIL patients, comparing the results with those obtained for twenty-two AD patients and seventeen control subjects.

## 2. Patients and Methods

After obtaining informed consent, we measured CSF A*β*42, t-tau, and p-tau levels in CADASIL patients, AD patients, and control subjects. 

CADASIL group included ten genetically confirmed patients (age range 49–66 years), followed in our department and already described in previous works by our group [22, 23]. Patients have been enrolled in a study on CSF biomarkers for the early diagnosis of dementia approved by local ethical committee. Detailed demographic data and molecular features of CADASIL patients are reported in Table 1.

The AD group consists of twenty-two patients with probable AD (age range 58–74 years) diagnosed in our department according to the NIN-CDS-ADRDA criteria [24]. AD patients with more than one cardiovascular risk factor and patient with even 1-2 white matter lacunes were excluded. None of the AD patients was under treatment for dementia at the time of lumbar puncture.

The control group included seventeen age-matched subjects with polyneuropathy and without signs of cognitive decline or central nervous system lesions. 

Overall cognitive performance was evaluated in all three groups by Mini-Mental-State Examination (MMSE). MMSE scores below 26 indicate the presence of cognitive impairment. 

CSF was obtained by standard procedures, collected in polypropylene tubes and immediately centrifuged. All blood contamination-free samples were stored at −80°C until assay, CSF levels of A*β*42, t-tau, and p-tau were determined by sensitive sandwich enzyme-linked immunoassay ELISA: Innotest *β*-amyloid 1-42, Innotest hTAU-Ag, and Innotest Phospho-tau 181p (specific for tau proteins phosphorylated at threonine 181) (Innogenetics, Ghent, Belgium). All determinations were done in duplicate.

## 3. Statistical Analysis

The nonparametric test of Kruskal-Wallis was used instead of the Analysis of Variance (ANOVA) because Levene's test rejected the hypothesis of equality of sample variances. Pairwise group comparisons were then made using the nonparametric test of Mann-Whitney with Bonferroni correction of type-I error probability.

A statistical significance level of 95% (type-I error probability, *P*, less than .05) was considered for all tests.

Statistical analysis was executed with the statistical package SPSS 10.

## 4. Results

**Figure 1:**
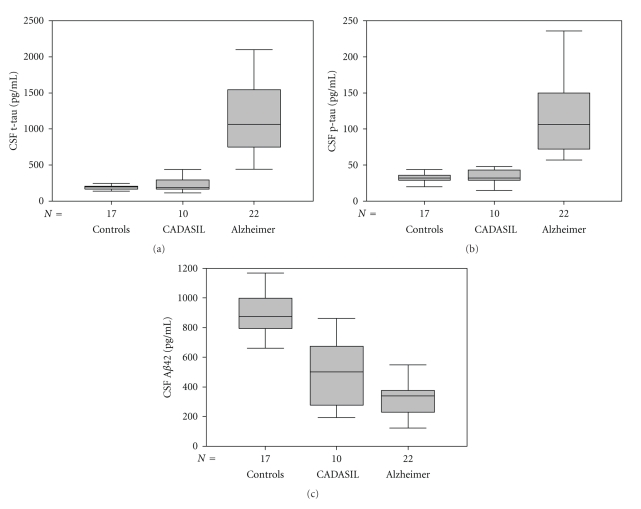
Box plots of CADASIL and Alzheimer patients and control subjects. They include median (horizontal line within box), interquartile interval, that is, 25th to 75th percentile (grey box) and range of variation (whiskers): (a) t-tau data, (b) p-tau data, and (c) A *β*_1-42_ data.

Descriptive statistics of clinical features and CSF levels of A*β*42, t-tau, and p-tau, including median and interquartile distance (25th to 75th percentile), for CADASIL, Alzheimer patients, and controls are reported at the bottom of Table 1. Figures 1(a)–1(c) supply a box plot representation of CSF grouped data.

*A*β**42. CSF A*β*42 levels were significantly lower both in CADASIL and in Alzheimer patients than in controls (Kruskal-Wallis and Bonferroni-corrected Mann-Whitney tests, *P* < .05). However CSF A*β*42 levels in CADASIL did not significantly differ from those in Alzheimer (Bonferroni-corrected Mann-Whitney test, *P* > .05). Group differences are illustrated in Figure 1(a): grey boxes, which represent group interquartile intervals (25th to 75th percentiles), overlap only for CADASIL and Alzheimer data.

*t-tau and p-tau*. Statistically significant higher values were found in Alzheimer with respect to both control and CADASIL subjects (Kruskal-Wallis and Bonferroni-corrected Mann-Whitney tests, *P* < .05), while these last two groups are not result statistically different (Bonferroni-corrected Mann-Whitney test, *P* > .05). Figures 1(b) and 1(c) show clear differences.

## 5. Discussion

Many studies evaluated changes in CSF A*β*42 and tau protein in prodromal stages of AD or in other types of dementia. Tau is an axonal protein that binds to tubulin in microtubules, promoting their assembly and stability [25]. Elevated CSF levels of t-tau and p-tau have been interpreted as indicators of ongoing neuronal and axonal degeneration and/or the presence of neurofibrillary tangles in the brain [26]. *β*-amyloid (A*β*) peptides are a major component of amyloid plaques deposited in the brain of patients with different neurodegenerative diseases. Decreased CSF A*β*42 levels have been ascribed to accumulation in senile plaques [27]. However alternative mechanisms, such as formation of a CSF chaperon complex with high-affinity binding and epitope masking of A*β*42, have also been reported [28]. 

Low CSF A*β*42, high t-tau, and p-tau levels are the typical pattern in AD, whereas in VD, until now, results on CSF biomarkers are conflicting [17, 18]. 

Ethnic and methodological differences may account for this discrepancy, as high assay variability is present amongst laboratories using the same ELISA test [29]. However VD is a heterogeneous entity and different authors may study different subpopulations of patients, and it is difficult to assess the specificity of CSF biomarkers profile. Furthermore, as many patients with VD also show cholinergic lesions of aging and AD, possible overlaps of vascular pathology and AD cannot be ruled out [30–32].

In a previous study we reported significantly lower CSF A*β*42 levels in CADASIL patients than in controls whereas CSF t-tau and p-tau did not differ between the two groups [33]. 

In the present study the CSF biomarkers levels of the same CADASIL patients have been compared with those of AD patients showing an altered profile in CADASIL patients: A*β*42 levels were markedly decreased and considerably overlapped with AD, whereas t-tau and p-tau levels were normal and significantly different with respect to AD. 

Recent studies on CSF biomarkers profile in sporadic VD showed a clear decrease of A*β*42 levels [19]. The mechanism of decreased CSF A*β*42 levels in cerebrovascular disease is unclear however the presence of these features in sporadic VD and in patients with a young-onset vascular disorder like CADASIL suggests that altered CSF A*β*42 levels may be related to subcortical vascular lesions and independent from an age- and AD-related pathology. Recently, a link between white matter lesions and low CSF A*β*42 has also been reported [34]. Moreover, the significant overlap of CSF A*β*42 levels in a pure VD and AD suggests to rule out any possible correlation between decreased CSF A*β*42 levels and accumulation in senile plaques and strengthens the most recent hypotheses about alternative mechanisms of A*β*42 reduction [28]. 

Normal t-tau and p-tau CSF levels we found in CADASIL contrast with the majority of the results of other groups on t-tau and p-tau values in sporadic VD. However, in the latter group of patients the possible presence of additional neuropathological changes associated with age and AD cannot be ruled out. Since CADASIL represents a model of pure VD, we can argue that t-tau and p-tau CSF levels in CADASIL patients closely reflect a pathological condition almost exclusively due to cerebrovascular features. We can thus suggest that CSF neurochemical phenotypes, especially t-tau and p-tau levels, sufficiently discriminate between AD and VD.

In conclusion, although our little sample size can reduce the statistical power of our results, significant differences in CSF biomarkers profile between pure VD, AD, and controls were found. These data support the use of CSF A*β*42, t-tau, and p-tau levels in the differential diagnosis of VD and AD.

 In any case, this study represents a preliminary investigation whose statistical results should be confirmed by further examinations on larger data samples.

## Figures and Tables

**Table 1:** Clinical and molecular features of CADASIL patients.

*n*°	CADASIL patients	age	sex	MMSE	mutation	CSF t-tau (pg/ml)	CSF A*β*_1-42_ (pg/ml)	CSF p-tau (pg/ml)
1	PV	66	M	24	r207c eter ex4	200	307	40
2	GP	53	F	19	r207c eter ex4	205	193	30
3	MM	50	M	25	r207c eter ex4	174	275	32
4	BAS	52	M	23	r607c eter ex11	295	487	48
5	SS	57	M	15	r607c eter ex11	170	520	32
6	TA	49	M	21	r1076c eter ex20	296	675	45
7	DPM	58	F	29	frame shift aa127–158 stop codon aa159 ex4	438	746	43
8	JF	49	M	24	g528c eter ex11	166	585	29
9	SME	67	M	25	r1076c eter ex20	120	863	27
10	FUS	39	M	23	R332C ex 6	114	244	15
CADASIL patients (10)		54±8,4^a^	2F/8M^**a**^	23±3,8^a^		187 [155–295]^b^	504 [267–693]^b^	32 [28–43]^b^
Alzheimer patients (22)		66±8,4^a^	15F/7M^a^	18±4,6^a^		1063 [748–1582]^b^	340 [225–378]^b^	106 [70–151]^b^
controls (17)		58±9,9^a^	11F/6M^a^	30±2,2^a^		197 [167–210]^b^	875 [793–1024]^b^	32 [26–38]^b^

MMSE: minimental state examination; ^a^values are expressed as mean ± S.D.; ^b^values are expressed as median [25th–75th percentile].
